# Prion protein facilitates retinal iron uptake and is cleaved at the β-site: Implications for retinal iron homeostasis in prion disorders

**DOI:** 10.1038/s41598-017-08821-1

**Published:** 2017-08-29

**Authors:** Abhishek Asthana, Shounak Baksi, Ajay Ashok, Shilpita Karmakar, Najiba Mammadova, Robyn Kokemuller, Mary Heather Greenlee, Qingzhong Kong, Neena Singh

**Affiliations:** 1 Department of Pathology, School of Medicine, Case Western Reserve University, Cleveland, Ohio, 44106 USA; 2 Department of Biomedical Sciences, Iowa State University College of Veterinary Medicine, Ames, Iowa 50010 USA

## Abstract

Prion disease-associated retinal degeneration is attributed to PrP-scrapie (PrP^Sc^), a misfolded isoform of prion protein (PrP^C^) that accumulates in the neuroretina. However, a lack of temporal and spatial correlation between PrP^Sc^ and cytotoxicity suggests the contribution of host factors. We report retinal iron dyshomeostasis as one such factor. PrP^C^ is expressed on the basolateral membrane of retinal-pigment-epithelial (RPE) cells, where it mediates uptake of iron by the neuroretina. Accordingly, the neuroretina of PrP-knock-out mice is iron-deficient. In RPE19 cells, silencing of PrP^C^ decreases ferritin while over-expression upregulates ferritin and divalent-metal-transporter-1 (DMT-1), indicating PrP^C^-mediated iron uptake through DMT-1. Polarization of RPE19 cells results in upregulation of ferritin by ~10-fold and β-cleavage of PrP^C^, the latter likely to block further uptake of iron due to cleavage of the ferrireductase domain. A similar β-cleavage of PrP^C^ is observed in mouse retinal lysates. Scrapie infection causes PrP^Sc^ accumulation and microglial activation, and surprisingly, upregulation of transferrin despite increased levels of ferritin. Notably, detergent-insoluble ferritin accumulates in RPE cells and correlates temporally with microglial activation, not PrP^Sc^ accumulation, suggesting that impaired uptake of iron by PrP^Sc^ combined with inflammation results in retinal iron-dyshomeostasis, a potentially toxic host response contributing to prion disease-associated pathology.

## Introduction

Prion disorders are a group of neurodegenerative conditions of humans and animals resulting from the misfolding of a normal cell surface glycoprotein, the prion protein (PrP^C^), from a mainly α-helical to a β-sheet rich conformation known as PrP-scrapie (PrP^Sc^). Accumulation of PrP^Sc^ in the brain parenchyma is believed to be the principal cause of neurotoxicity in all prion disorders. The underlying mechanism is complex, and involves the expression of PrP^C^ on host cells for PrP^Sc^-induced cytotoxicity^1, 2^. Though useful, this information is of little help in developing therapeutic options. Recent reports indicating a temporal and spatial disconnect between PrP^Sc^ accumulation and neurotoxicity suggest the contribution of region and cell-specific host factors, an observation likely to provide alternative strategies for mitigating PrP^Sc^-induced cytotoxicity^3, 4^. Among these, iron dyshomeostasis and neuro-inflammation have long been suspected as major players in the neurotoxicity associated with prion and other neurodegenerative conditions^5–9^. A partial success in alleviating the symptoms of Alzheimer’s disease, Parkinson’s disease, and aceruloplasminemia with iron chelators is encouraging^10, 11^. However, a deeper understanding of the nature and cause of iron dyshomeostasis in each disorder is necessary to fully utilize this option.

In prion disorders, brain iron dyshomeostasis is believed to result from loss of function of PrP^C^ in iron uptake combined with sequestration of iron in dysfunctional ferritin, creating functional iron deficiency^12, 13^. A phenotype of relative iron deficiency despite sufficient or increased total iron has been demonstrated in biopsy and autopsy brain samples from sporadic Creutzfeldt-Jakob disease (sCJD) cases, the most common human prion disorder, and brain tissue harvested from scrapie-infected hamsters and mice during prion disease progression^12, 13^. Neuro-inflammation associated with PrP^Sc^ accumulation^4^ is likely to exacerbate the functional iron deficiency by sequestering iron within cells, a well-known response to chronic inflammation^14^. Since accumulation of PrP^Sc^ involves conversion of PrP^C^ to a dysfunctional, aggregated form that induces microglial activation^4, 15, 16^, it is likely that loss of function of PrP^C^ in iron uptake, aggregation of ferritin, and PrP^Sc^-induced inflammation evolve simultaneously, making it difficult to identify the contribution of each to iron dyshomeostasis. To differentiate between these processes, it is important to understand the functional role of PrP^C^ in iron uptake, and the effect of PrP^Sc^ and inflammation on iron metabolism at the cellular and organ level *in vivo* in a relatively simple model where temporal and spatial correlation between the appearance of PrP^Sc^, microglial activation, and accumulation of ferritin can be mapped during disease progression.

The neuroretina provides such an opportunity, and was used in this study to understand and differentiate between the above processes. The neuroretina is separated from the systemic circulation by the outer and inner blood-retinal barriers, much like the blood-cerebrospinal fluid (CSF) and blood-brain barrier (BBB) in the brain. The outer blood-retinal barrier comprises of a monolayer of retinal pigment epithelial (RPE) cells that regulate the transport of iron and other nutrients from choroidal blood to the neuroretina. The inner blood-retinal barrier is formed by a monolayer of endothelial cells lining the capillaries that mediate the transport of nutrients from capillary blood to the neuroretina as in the BBB^17^. Furthermore, the neuroretina accumulates PrP^Sc^ and undergoes degeneration in sCJD and scrapie-infected animal models^3, 16, 18–20^, providing an experimentally manipulable model to understand the role of PrP^C^ in iron transport and the role of PrP^Sc^ and inflammation in retinal iron homeostasis and neuroretinal degeneration.

We report that PrP^C^ is expressed on the basolateral (BL) membrane of RPE cells where it regulates the transport of iron from choroidal capillaries to the neuroretina. Surprisingly, majority of PrP^C^ is cleaved at the β-site in these cells, suggesting a regulatory role in iron uptake or a presently unknown cell-specific functional role. Scrapie infection results in the accumulation of detergent-insoluble ferritin that correlates temporally with microglial activation, suggesting a prominent role of inflammation in prion disease associated retinal iron dyshomeostasis. These observations are discussed with reference to the physiological function of PrP^C^ in retinal iron uptake, and implications of PrP^Sc^ accumulation on retinal iron homeostasis.

## Results

### PrP^C^ is expressed on the basolateral membrane of RPE cells and mediates transport of iron to the neuroretina

**Figure 1:**
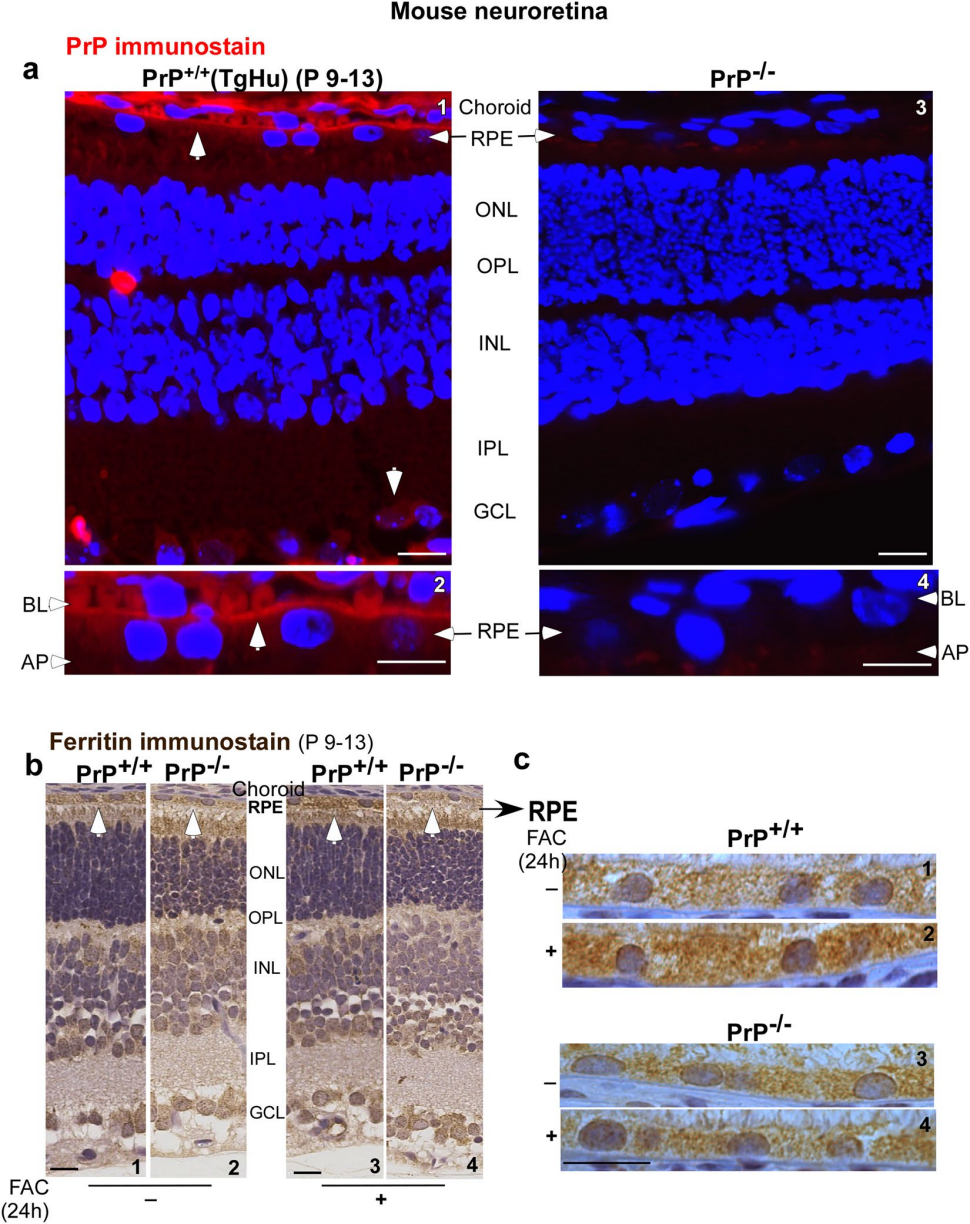
Distribution of PrP^C^ and ferritin in the neuroretina of PrP^+/+^ and PrP^−/−^ mice. (**a**) Immunoreaction of retinal sections from 9–13 day old PrP^+/+^ (TgHu) mice with 3F4 revealed a strong reaction for PrP^C^ on the BL membrane of RPE cells in addition to its reported expression on ganglion cells (panels 1 & 2). No reaction was detected in PrP^−/−^ samples processed in parallel (panels 3 & 4). **(b**) Immunoreaction for ferritin shows stronger reactivity in the RPE cell layer of PrP^+/+^ relative to PrP^−/−^ sample (panel 1 vs. 2). Systemic iron overload upregulates ferritin mainly in the RPE cell layer of PrP^+/+^ samples, not in PrP^−/−^ samples (panel 3 vs. 4 & 2 vs.4). **(c)** Higher magnification images of the RPE cell layer show a prominent increase in intracellular ferritin in PrP^+/+^ samples following systemic iron overload (panel 1 vs. 2). PrP^−/−^ samples show minimal change (panel 3 vs. 4). RPE: retinal pigment epithelium; ONL: outer nuclear layer; OPL: outer plexiform layer; INL: inner nuclear layer; IPL: inner plexiform layer; GCL: ganglion cell layer: BL: basolateral; AP: apical. Scale bar 20 μm.

The expression of PrP^C^ in the neuroretina is based mainly on histochemical studies designed to detect PrP^Sc^. Based on these and other studies, PrP^C^ has been detected in the inner and outer plexiform layers, inner segments of photoreceptors, and the ganglion and nerve fiber cell layers^21, 22^. To improve the sensitivity of detection, we immunostained retinal sections of eyes harvested from transgenic mice over-expressing human PrP^C^ (PrP^+/+^ (TgHu)) in PrP-null background^23^ with 3F4, a highly sensitive and specific monoclonal antibody that reacts with human and hamster PrP^C^, not with mouse PrP^C^. A strong immunoreaction was observed on the BL surface of the RPE cell layer in addition to its reported expression in other areas (Fig. 1a, panels 1 & 2, arrows). Similarly processed samples from PrP^−/−^ mice showed no reactivity with 3F4 (Fig. 1a, panels 3 & 4).

The RPE cells form a polarized monolayer with tight junctions with the BL membrane facing the Bruch’s membrane that is juxtaposed to the fenestrated choroidal capillaries. The apical (AP) membrane of RPE cells faces the neuroretina. As in other polarized cells, each domain is equipped to perform its specific function. The BL domain of RPE cells performs the essential function of regulating the uptake and efflux of essential nutrients to and from the neuroretina^17^. Since PrP^C^ is known to facilitate iron uptake, its expression on the BL membrane of RPE cells suggested a similar role at this site. To evaluate this possibility, wild-type (PrP^+/+^) and matched PrP^−/−^ mice were injected intraperitoneally with 70 µg/22 g mouse weight of ferric ammonium citrate (FAC) to create systemic iron over-load. Under these conditions serum transferrin (Tf) is fully saturated with iron, and the pool of non-transferrin bound iron (NTBI) increases significantly^24^. Following a chase of 24 hours, mice were euthanized, capillary blood was flushed with PBS, and harvested eyes were processed for immunohistochemistry. Retinal sections from each set of mice were exposed to ferritin-specific primary antibody that recognized both H- and L-chain ferritin sub-units, followed by peroxidase-conjugated secondary antibody. The reaction was developed using DAB as the substrate, resulting in a brown precipitate. Each set of sections was processed in parallel to eliminate experimental error.

At steady state, PrP^−/−^ samples were less immunoreactive for ferritin in the RPE cell layer and other cell types relative to matched PrP^+/+^ controls (Fig. 1b, panels 1 & 2). Systemic iron over-load increased ferritin reactivity in the neuroretina of PrP^+/+^ mice (Fig. 1b, panels 1 & 3), but had minimal effect on PrP^−/−^ mice (Fig. 1b, panels 2 & 4). The differential effect of systemic iron over-load in PrP^+/+^ and PrP^−/−^ samples is more obvious in the RPE cell layer (Fig. 1c, panels 1–4), suggesting a facilitative role of PrP^C^ in the uptake of both transferrin bound iron (Tf-Fe) and NTBI.

**Figure 2:**
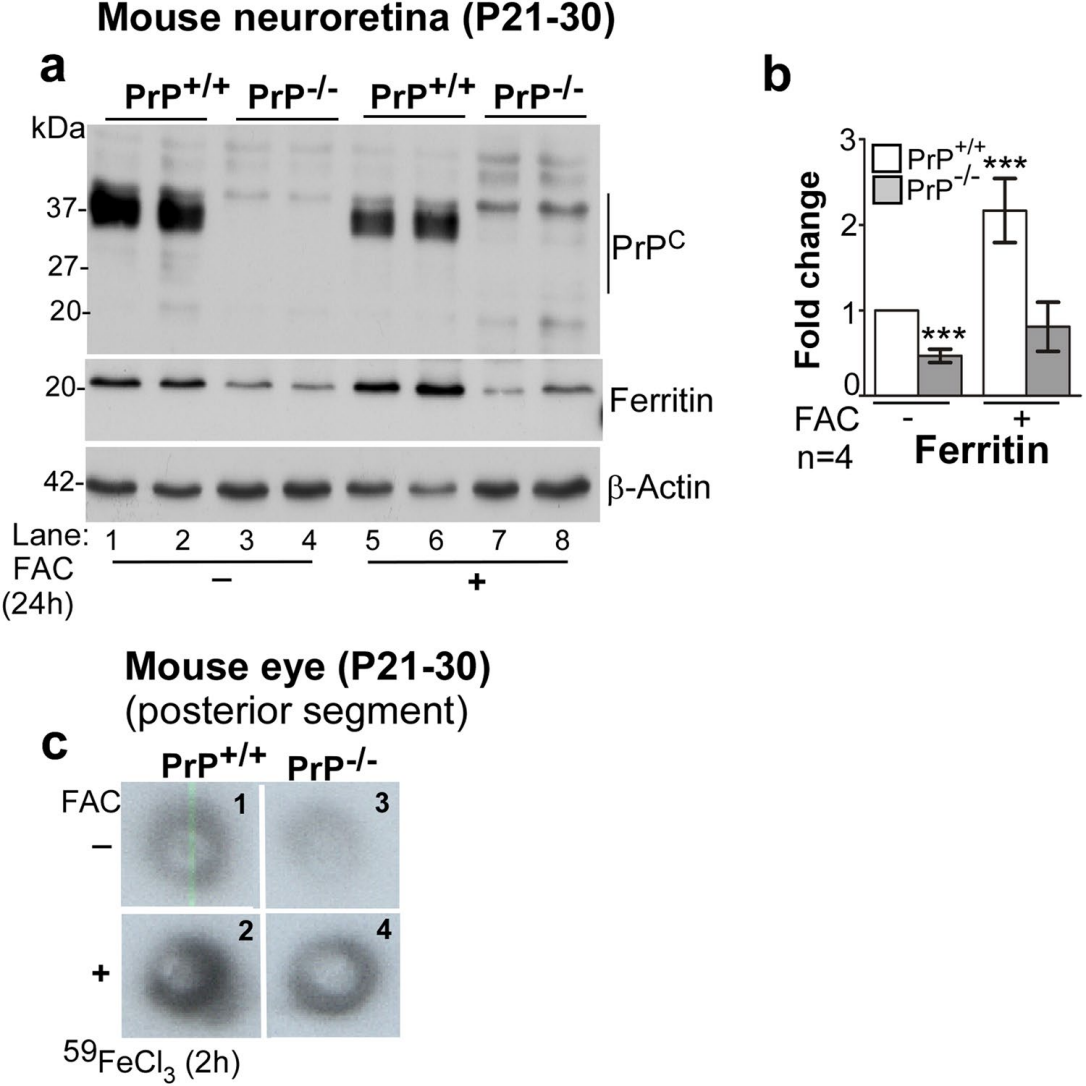
The neuroretina of PrP^−/−^ mice is iron deficient. (**a**) Probing of pooled retinal lysates for PrP^C^ with anti-C (8H4) shows the expected full-length glycoforms of PrP^C^ in PrP^+/+^ samples (lanes 1 & 2), and no signal in PrP^−/−^ samples as expected (lanes 3 & 4). Re-probing for ferritin shows significantly more signal in PrP^+/+^ relative to PrP^−/−^ samples (lanes 1 & 2 vs. 3 & 4; b). A similar analysis of neuroretinal samples from iron-overloaded mice shows significant upregulation of ferritin in PrP^+/+^ samples (lanes 1 & 2 vs. 5 & 6; b), and minimal change in PrP^−/−^ samples (lanes 3 & 4 vs. 7 & 8; b). **(b)** Densitometric analysis of protein bands after normalization with β-actin shows 0.5 fold less ferritin in PrP^−/−^ relative to PrP^+/+^ samples. Systemic iron overload causes 2-fold upregulation of ferritin in PrP^+/+^ samples, but minimal change in PrP^−/−^ samples. Values are mean ± SEM of the indicated n. ***p < 0.001. **(c)** Fluorograph of the posterior segment of eye shows less uptake of ^59^Fe in PrP^−/−^ relative to PrP^+/+^ samples (panel 1 vs. 3). Systemic iron overload increases ^59^Fe uptake in both PrP^+/+^ and PrP^−/−^ samples (panels 1 & 3 vs. 2 & 4), but the increase is less in PrP^−/−^ relative to PrP^+/+^ controls (panel 2 vs. 4).

The above observations were confirmed by subjecting retinal lysates isolated from control and iron-overloaded PrP^+/+^ and PrP^−/−^ mice to Western blotting. Probing with 8H4 antibody that reacts with mouse (and human) PrP^C^ revealed the expected glycoforms in PrP^+/+^ samples, and their complete absence in PrP^−/−^ samples (Fig. 2a, lanes 1–8). Subsequent probing for ferritin revealed significantly less expression in PrP^−/−^ samples relative to PrP^+/+^ controls at steady state (Fig. 2a, lanes 1 & 2 vs. 3 & 4; Fig. 2b). Iron overloading upregulated ferritin expression in PrP^+/+^ samples (Fig. 2a, lanes 1 & 2 vs. 5 & 6; Fig. 2b), but had minimal effect on matched PrP^−/−^ samples (Fig. 2a, lanes 3 & 4 vs. 7 & 8; Fig. 2b) even though both mouse lines show systemic iron overload under these conditions^25^.

Although ferritin expression is a valuable indicator of cellular iron levels in the absence of other confounding factors such as inflammation, we confirmed these observations by quantifying the uptake of radiolabeled iron (^59^Fe). To radiolabel mainly ^59^Fe-Tf, mice were injected with ^59^FeCl_3_ intraperitoneally. Since Tf has high affinity for iron, most of the injected ^59^Fe binds circulating Tf. To radiolabel NTBI specifically, mice were over-loaded with unlabeled FAC as above to saturate circulating Tf prior to injecting ^59^FeCl_3_^24^. Uptake of ^59^Fe by the whole eye was quantified in a γ-counter. In addition, the posterior segment of eye was dissected and exposed to X-ray film. Uptake of both ^59^Fe-Tf and ^59^Fe-NTBI was lower in PrP^−/−^ samples relative to PrP^+/+^ controls (Fig. 2c, panels 1 & 2 vs. 3 & 4). Although iron over-loading increased the uptake of ^59^Fe-NTBI in both mouse lines, levels in PrP^−/−^ samples remained lower than PrP^+/+^ controls.

Together, the above results demonstrate that PrP^C^ facilitates uptake of iron from the choroidal capillaries to the neuroretina. Subsequent studies were designed to confirm this phenomenon and understand the underlying mechanism in cell lines that allow greater flexibility of experimental manipulation.

### PrP^C^ facilitates uptake of iron by RPE19 cells through DMT-1

**Figure 3:**
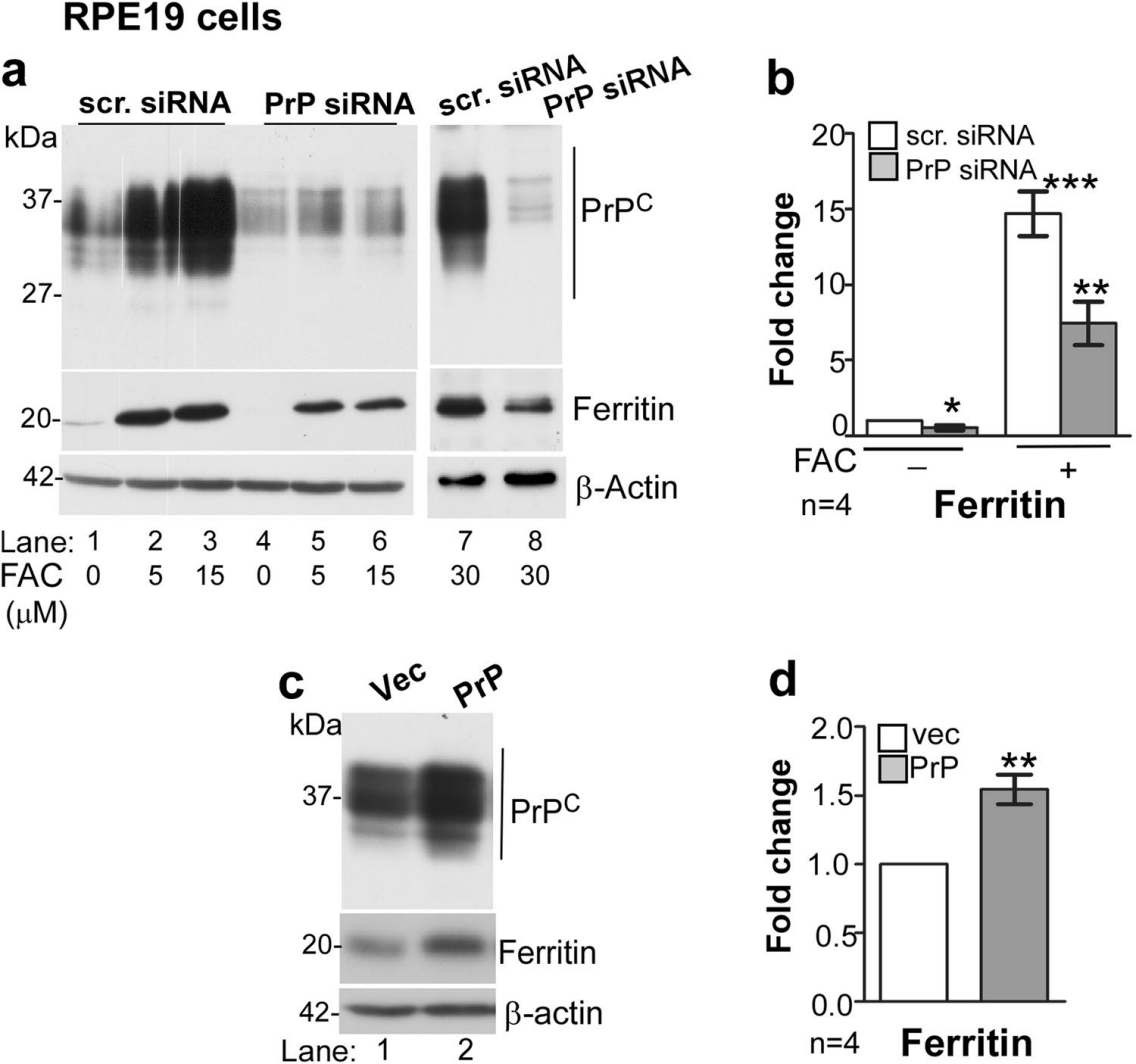
PrP^C^ influences ferritin levels in RPE19 cells. (**a**) Down-regulation of PrP^C^ with siRNA reduces ferritin expression in untreated (lanes 1 vs. 4) and FAC treated cells (lanes 2 & 3 vs. 5 & 6; lane 7 vs. 8) (**b**) Densitometric analysis of protein bands after normalization with β-actin shows 0.5-fold reduction in ferritin after down-regulation of PrP^C^. Exposure to FAC upregulates ferritin by ~14-fold in controls, but only 6–7 fold in cells where PrP^C^ is downregulated. (**c** and **d**) Over-expression of PrP^C^ results in upregulation of ferritin by 1.5 fold (lane 1 vs. 2; d). β-actin served as a loading control. Values are mean ± SEM of the indicated n. *p < 0.05, **p < 0.01, ***p < 0.001.

To further confirm the functional role of PrP^C^ in iron uptake, we used a human RPE cell line (RPE19) that expresses abundant amounts of PrP^C^. Silencing of PrP^C^ with RNAi resulted in down-regulation of ferritin, and this effect was amplified in the presence of extracellular iron (Fig. 3a, lanes 1 vs. 4, lanes 2 & 3 vs. 5 & 6, lane 7 vs. 8; Fig. 3b). Likewise, over-expression of PrP^C^ caused significant upregulation of ferritin (Fig. 3c, lanes 1 & 2; Fig. 3d), supporting a facilitative role of PrP^C^ in iron uptake.

**Figure 4:**
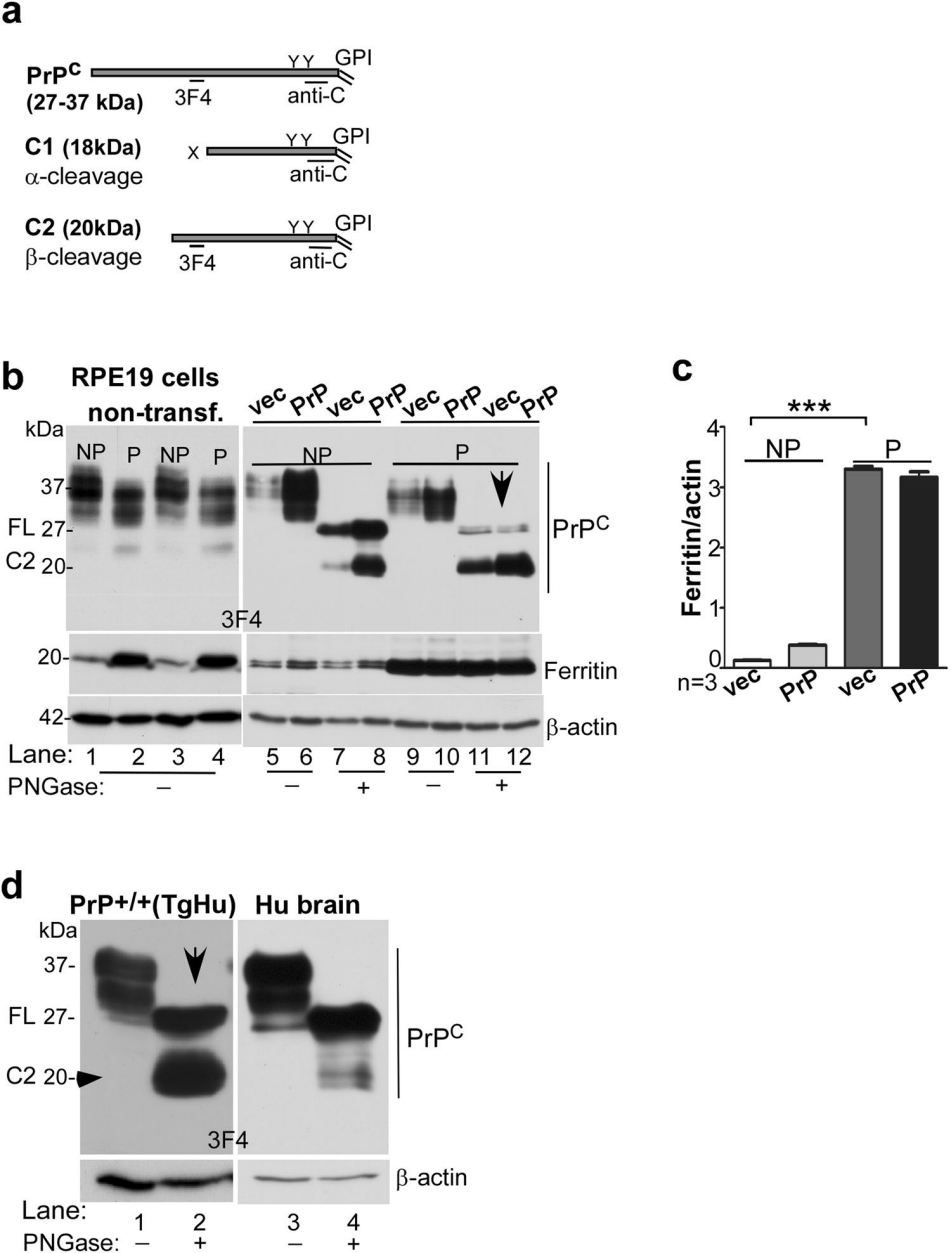
Polarization of RPE cells results in upregulation of ferritin and β-cleavage of PrP^C^. (**a**) Schematic representation of full length (FL), α-cleaved (C1), and β-cleaved (C2) forms of PrP^C^ and antibody reactivity against 3F4 and anti-C (8H4) epitopes. (**b**) Probing of lysates from non-transfected and PrP^C^ expressing RPE19 cells with 3F4 shows cleavage of PrP^C^ at the β-site following polarization, resulting in the generation of C2 (lanes 1 & 3 vs. 2 & 4). The change in the ratio of full-length (FL) vs. β-cleaved (C2) form of PrP^C^ is more obvious in deglycosylated samples (lanes 5–8 vs. 9–12). Re-probing for ferritin shows upregulation in PrP^C^-expressing cells relative to vector controls (lanes 5 & 7 vs. 6 & 8), and a dramatic increase in both cell lines following polarization (lanes 1 & 3 vs. 2 & 4, lanes 5–8 vs. 9–12). NP: non-polarized; P: polarized. **(c)** Densitometric analysis of protein bands following normalization with β-actin shows ~10 fold increase in ferritin following polarization in both vector and PrP^C^-expressing cells. Values are mean ± SEM of the indicated n. ***p < 0.001. (**d**) Probing of neuroretinal lysates from PrP^+/+^ (TgHu) mice and human brain for PrP^C^ shows β-cleavage of ~70% of PrP^C^ in the neuroretina relative to ~10% in brain samples.

To evaluate whether PrP^C^ undergoes similar post-translational processing in RPE cells as in other cell lines, lysates of vector and PrP^C^-transfected cells were subjected to deglycosylation and analyzed by Western blotting. Two different antibodies were used to differentiate between different processing events of PrP^C^; anti-C (or 8H4) that reacts with full-length and α- and β-cleaved C-terminal fragments of PrP^C^, and 3F4 that recognizes full-length and only β-cleaved fragment of PrP^C^ (Fig. 4a).

Biochemical evaluation of lysates prepared from non-polarized (NP) and polarized (P) cells revealed a robust increase in ferritin expression by ~10-fold upon polarization (Fig. 4b, lanes 1 vs. 2, 3 vs. 4, & lanes 5–8 vs. 9–12; Fig. 4c). Although the positive influence of PrP^C^ on ferritin was observed in non-polarized cells (Fig. 4b, lanes 5 vs. 6 & 7 vs. 8), it was difficult to discern in polarized cells likely due to the strong reactivity of ferritin bands in these samples (Fig. 4b, lanes 9–12; Fig. 4c).

Apart from the unexpected increase in ferritin expression, almost all of PrP^C^ was cleaved at the β-site on polarization of RPE19 cells (Fig. 4b, lanes 7 & 8 vs. 11 & 12). This was a surprising observation since majority of PrP^C^ undergoes α-cleavage during recycling from the plasma membrane under physiological conditions^26–28^, and cleavage at the β-site is typical of the disease-associated PrP^Sc^ isoform^26^. A similar β-cleavage of PrP^C^ was observed in TgHu mouse neuroretinal lysates (Fig. 4d, lanes 1 & 2), confirming that this observation is not an artifact of the cell line. Brain lysates processed in parallel showed minimal cleavage of PrP^C^ at the β-site as expected (Fig. 4d, lanes 3 & 4).

**Figure 5:**
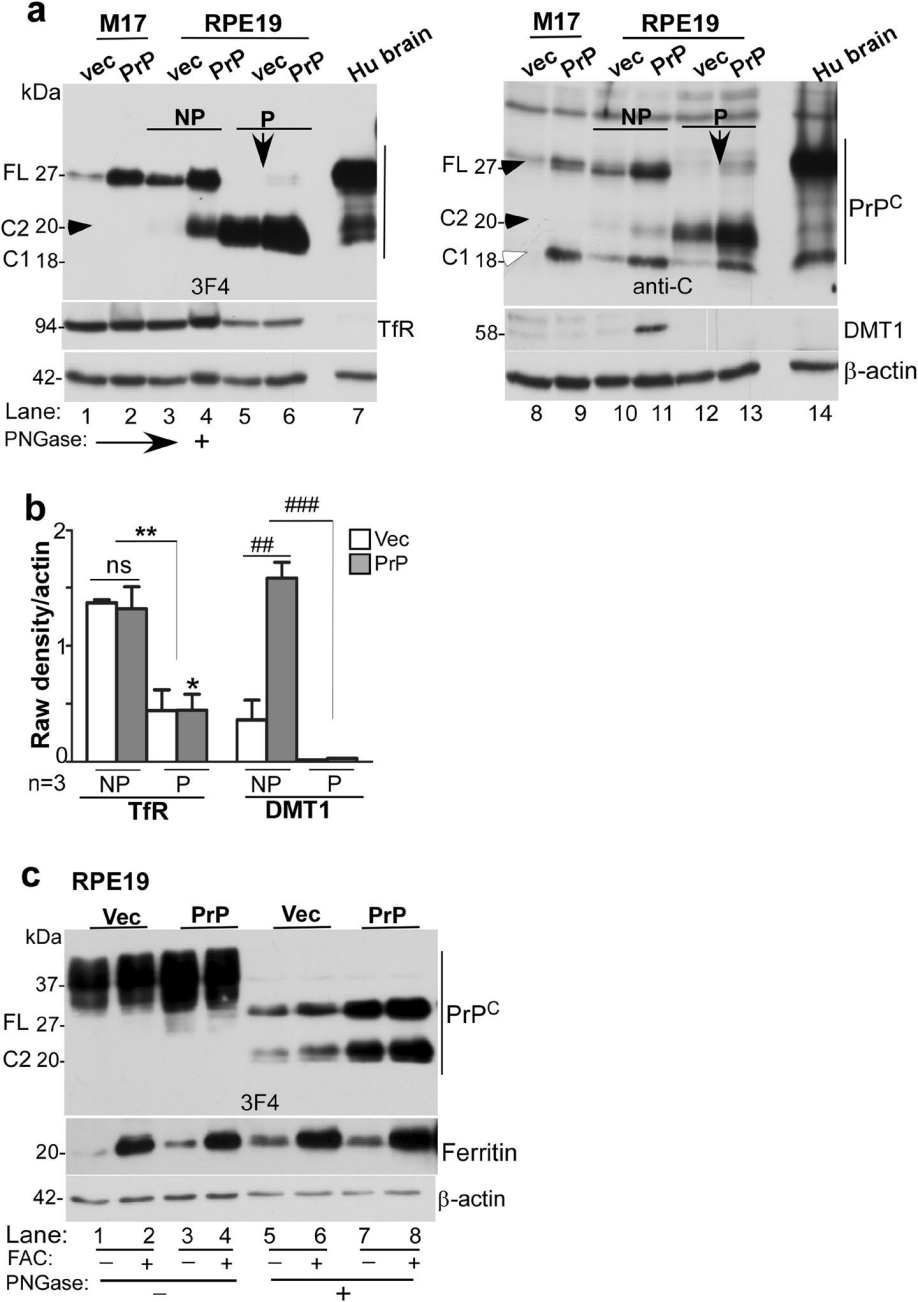
β-cleavage of PrP^C^ modulates intracellular iron and is unique to polarized RPE cells. (**a**) Deglycosylated lysates from vector and PrP^C^-expressing M17 and RPE cells and human brain were probed with PrP-specific antibodies 3F4 and anti-C to estimate the relative abundance of full-length (FL), α-cleaved (C1), and β-cleaved (C2) forms in each sample. Probing with 3F4 that reacts with FL and C2 (Fig. 4a) reveals undetectable levels of C2 in M17 cells even after over-expressing PrP^C^ (lanes 1 & 2). RPE cells, on the other hand, show significantly more C2 in non-polarized cells (lanes 3 & 4), and almost complete cleavage of FL to the C2 form following polarization (lanes 5 & 6). The brain sample shows minimal levels of C2 as in M17 cells (lane 7). Probing with anti-C antibody that reacts with FL, C1, and C2 (Fig. 4a) shows FL and C1 in M17 and brain lysates (lanes 8, 9, 14), and mainly C2 in RPE samples (lanes 10–13). Re-probing for TfR shows down-regulation following polarization of RPE cells (lanes 3 & 4 vs. 5 & 6). Re-probing for DMT-1, on the other hand, shows up-regulation in PrP^C^-expressing non-polarized RPE cells (lanes 10 & 11), and down-regulation following polarization (lanes 10 & 11 vs. 12 & 13). **(b)** Densitometric analysis of protein bands after normalization with β-actin shows downregulation of TfR following polarization of RPE cells to less than half, and upregulation of DMT-1 in PrP^C^-expressing non-polarized cells by ~3-fold. DMT-1 expression is minimal in polarized RPE cells. Values are mean ± SEM of the indicated n. ns, not significant, *p < 0.05, **p < 0.01, ^##^p < 0.01, ^###^p < 0.001. **(c)** Exposure of non-polarized RPE cells to FAC increases the ratio of C2 vs. FL that is more evident following deglycosylation (lanes 5 & 7 vs. 6 & 8; d). Re-probing for ferritin shows a significant increase following FAC treatment as expected (lanes 2, 4, 6, 8).

Further comparison with M17 cells, a neuroblastoma cell line, revealed similar results. Deglycosylation of lysates prepared from M17 and RPE19 cells followed by Western blotting with PrP-specific antibodies that bind specifically to the full-length and β-cleaved fragment of PrP^C^ (3F4), or full-length and both β- and α-cleaved fragments of PrP^C^ (anti-C) confirmed the above results. In M17 cells, majority of PrP^C^ was cleaved at the α-site (Fig. 5a, lanes 1, 2, 8, 9). In non-polarized RPE19 cells, the β-cleaved form was more prominent (Fig. 5a, lanes 3, 4, 10, 11). In polarized RPE19 cells, on the other hand, most of the PrP^C^ was in the β-cleaved form (Fig. 5a, lanes 5, 6, 12, 13). In addition, the expression of TfR was significantly reduced in polarized RPE19 cells, indicating increase in intracellular iron following polarization (Fig. 5a, lanes 3 & 4 vs. 5 & 6; Fig. 5b). These observations are consistent with significant increase in ferritin following polarization as observed in Fig. 4 above. The expression of DMT-1 was upregulated in non-polarized cells transfected with PrP^C^ and down-regulated following polarization, indicating that PrP^C^ facilitates iron uptake through DMT-1 (Fig. 5a, lanes 10 & 11 vs. 12 & 13; Fig. 5b). Exposure of RPE cells to exogenous iron increased β-cleavage of PrP^C^, suggesting that the cleavage is probably mediated by redox-active iron (Fig. 5c, lanes 1–8).

### Scrapie infection induces functional iron deficiency in the retina

**Figure 6:**
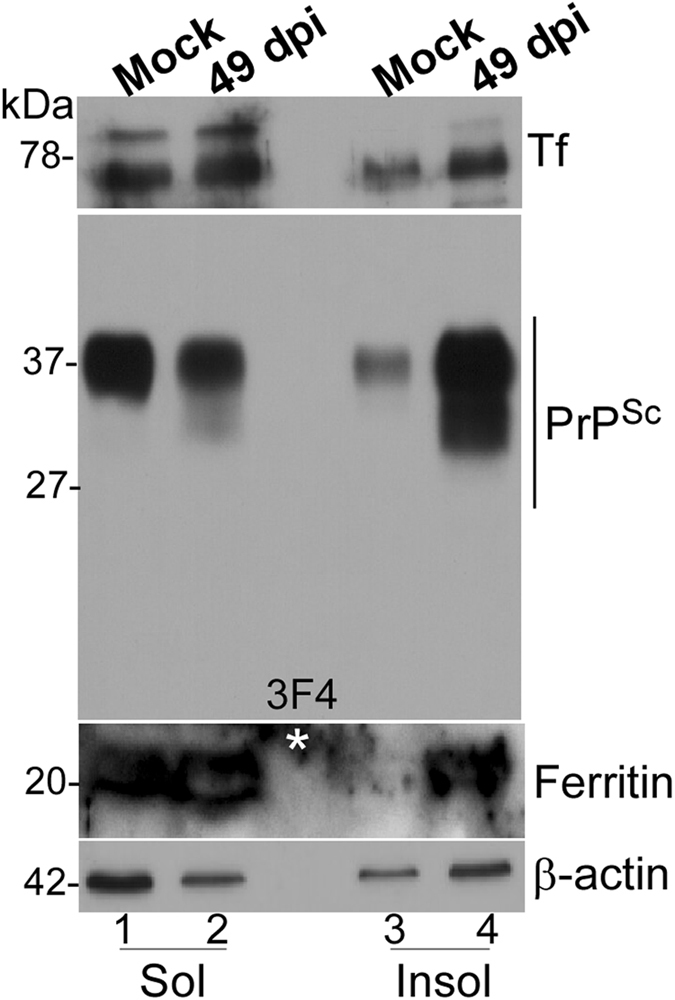
Scrapie infected hamsters show retinal iron imbalance. Neuroretinal lysates from mock and scrapie-infected hamsters were subjected to differential centrifugation to separate detergent-soluble and insoluble proteins, and processed for Western blotting. Probing for PrP reveals partitioning of most of the PrP^C^ from mock infected samples in the detergent-soluble phase (lanes 1 & 3), while the majority of PrP^Sc^ from infected samples partitions in the detergent-insoluble phase (lanes 2 & 4). Re-probing for Tf shows up-regulation in scrapie-infected samples, most of which partitions in the detergent-soluble phase (lanes 1–4). Re-probing for ferritin shows significant up-regulation in scrapie-infected samples, and partitioning of a significant amount in the detergent-insoluble phase (lanes 1–4; *non-specific reaction).

Since PrP^C^ mediates uptake of iron to the neuroretina, we next evaluated whether its conversion to PrP^Sc^ alters retinal iron homeostasis during scrapie infection. Thus, hamsters were injected with 263 K strain of scrapie intracerebrally (ic), and the eyes were harvested at 49 and 75 days post infection (dpi), and when clinical signs of scrapie were obvious (>80 days). Mock treated controls received normal brain homogenate instead. At the indicated times, one eye from each animal was dissected to harvest the neuroretina, and the other was fixed and processed for immunostaining. Evaluation of samples from 49 dpi samples by Western blotting showed relatively less reactivity for PrP^C^ in the soluble fraction relative to the control (Fig. 6, lanes 1 & 2), most of which partitioned in the detergent-insoluble fraction (Fig. 6, lanes 2 & 4). Most of the PrP^C^ from mock treated samples was recovered in the detergent-soluble fraction as expected (Fig. 6, lanes 1 & 3). Re-probing for Tf revealed reactivity in the detergent soluble fraction in both mock and scrapie-infected samples. Ferritin, on the other hand, partitioned mainly in the detergent insoluble fraction in scrapie-infected samples (Fig. 6, lanes 3 & 4). Surprisingly, both Tf and ferritin were upregulated in scrapie-infected samples, an unexpected observation since these proteins are regulated in a reciprocal manner to maintain cellular iron homeostasis (Fig. 6, lanes 1–4).

**Figure 7:**
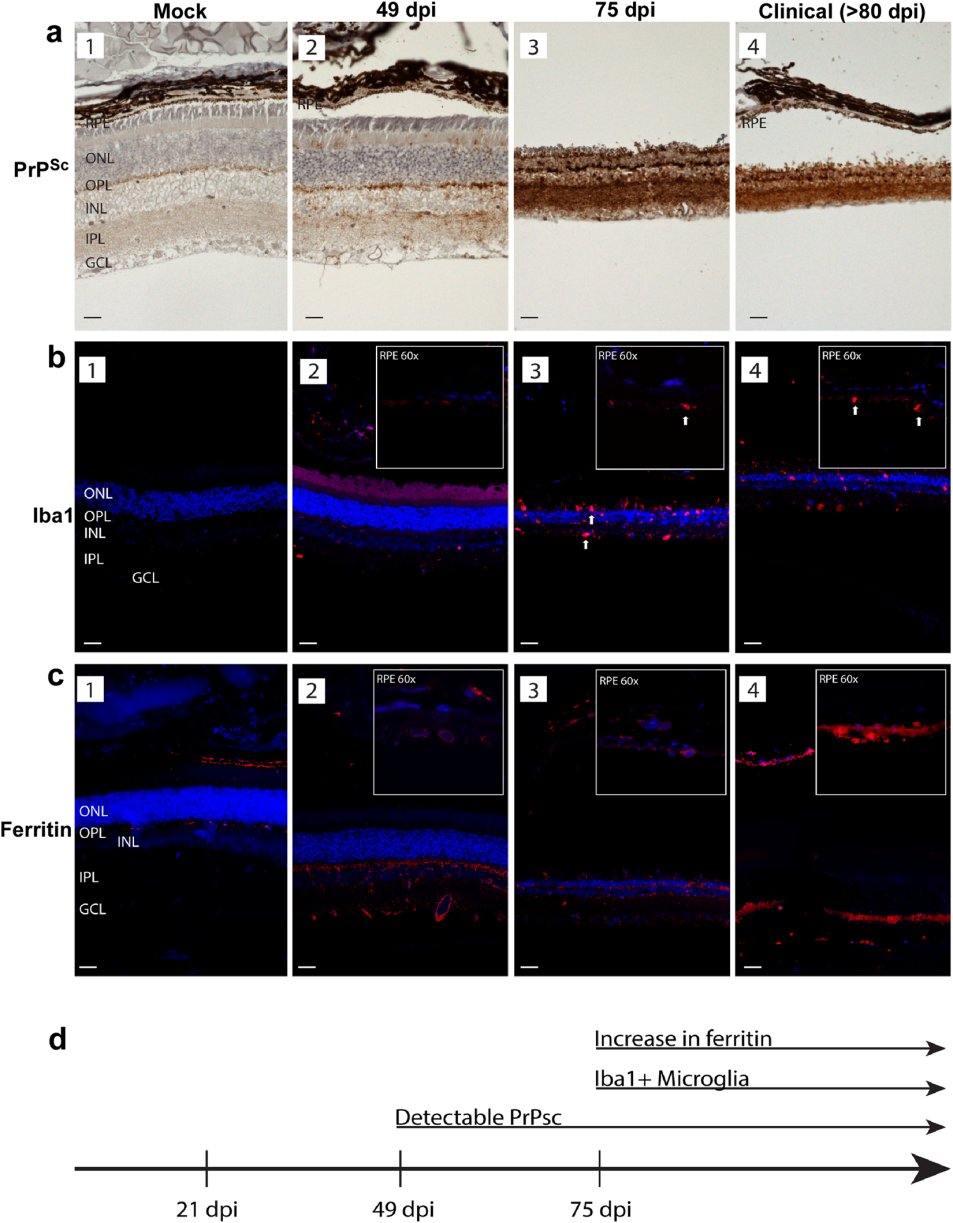
Distribution of PrP^Sc^, Iba1, and ferritin in hamster retinas. (**a**) Immunoreaction for PrP^Sc^ shows no reactivity in mock-treated control retinas (panel 1). PrP^Sc^ deposits are first evident at 49 dpi, and are localized to outer segments of photoreceptor cells, OPL, INL, IPL, and GCL (panel 2). Robust PrP^Sc^ deposits are seen throughout the retina at 75 dpi and in clinical samples (panels 3 & 4). (**b**) Immunoreaction for Iba1 shows minimal reactivity in RPE, OPL, and IPL in mock treated samples (panel 1). Prominent reactivity for Iba1 is first seen at 49 dpi (panel 2). Amoeboid microglia are evident throughout the retina, including the RPE cell layer in scrapie-infected samples (panels 3 & 4). Arrows indicating large cell bodies of activated, Iba1 + microglia at later time points (panels 3 & 4). (**c**) Immunoreaction for ferritin shows minimal reaction in mock-treated samples (panel 1). Increased perinuclear and cytoplasmic immunoreactivity for ferritin is first evident at 49dpi, and is localized to RPE, OPL, INL, IPL, and GCL (panels 2–4). High magnification (60X) inserts in the upper right hand corner show Iba1 and ferritin immunoreactivity in the RPE cell layer in scrapie-infected samples. Abbreviations: GCL: ganglion cell layer; IPL: inner plexiform layer; INL: inner nuclear layer; OPL: outer plexiform layer; ONL: outer nuclear layer; RPE: Retinal pigment epithelium. Scale bars: 10 μm. (**d**) The increase in ferritin reactivity occurs subsequent to PrP^Sc^ accumulation, and coincides with Iba1 reactivity.

To detect PrP^Sc^ by immunohistochemistry, retinal sections were autoclaved to denature PrP^Sc^ before reaction with PrP-specific antibody. This procedure precludes detection of PrP^C^ ^3^. Evaluation of mock-treated samples revealed normal retinal architecture and no specific reaction for PrP^Sc^ in the RPE cell layer, the ganglion cells, and inner (IPL) and outer plexiform (OPL) layers where normal PrP^C^ is expressed (Fig. 7a, panel 1). On the other hand, scrapie-infected samples showed increased reactivity for PrP and extensive destruction of the neuroretina with disease progression (Fig. 7a, panels 2–4). Immunoreactivity for Iba1, a marker for activated microglia, increased with disease progression, especially in the 75 dpi and clinically sick animals (Fig. 7b, panels 2–4). Likewise, immunoreactivity for ferritin increased with disease progression, and was most prominent in RPE and IPL cell layers (Fig. 7c, panels 2–4). In the RPE cells, ferritin showed an intracellular, punctate reaction (Fig. 7c, panel 4, inset). There was no obvious co-localization of reactivity for PrP^Sc^, Iba1, or ferritin. However, upregulation of ferritin coincided with the appearance of Iba1 on 75 dpi, ~25 days after the appearance of PrP^Sc^ (Fig. 7a–c; Fig. 7d).

## Discussion

This report highlights novel features of PrP^C^ in the neuroretina that shed light on its physiological function in iron uptake and the mechanism underlying prion disease-associated iron dyshomeostasis. PrP^C^ was expressed on the BL domain of RPE cells, where it facilitated transport of iron to the neuroretina. Surprisingly, 90% of PrP^C^ was cleaved at the β-site under iron-replete conditions, suggesting a regulatory role in iron uptake by shedding its ferrireductase domain. Experimental infection with scrapie resulted in the accumulation of PrP^Sc^ in the neuroretina as expected. However, the accompanying aggregation of ferritin and up regulation of transferrin was unexpected, and correlated temporally with microglial activation rather than PrP^Sc^ accumulation. These observations suggest that a combination of impaired uptake of iron by dysfunctional PrP^C^ (or PrP^Sc^) and inflammation-induced sequestration of iron in ferritin induce iron dyshomeostasis, a toxic host response that amplifies prion disease-associated cytotoxicity.

The polarized distribution of PrP^C^ on the BL domain of RPE cells was unexpected, and suggested a role in iron transport as in other cell types and mouse models^29–31^. RPE cells express Tf receptor (TfR) on the BL domain^32^ where it binds Tf-iron (Tf-Fe^3+^) in choroidal blood, and the Tf-Fe^3+^/TfR complex is endocytosed through clathrin-coated pits. Tf-bound Fe^3+^ is released in the acidic environment of endosomes, and the Tf/TfR complex is recycled back to the plasma membrane in a domain-specific manner. The released Fe^3+^ is reduced to Fe^2+^ by membrane-bound ferrireductase (FR) proteins such as Steap3 and PrP^C^ ^31, 33^, and Fe^2+^ is transported to the cytosol through DMT-1^34, 35^. Cytosolic iron is utilized by RPE cells for metabolic purposes, and exported to the neuroretina through an as yet undefined pathway because the only known iron exporter ferroportin (Fpn) is expressed on the BL domain^32^. An alternate hypothesis proposes transcytosis of Tf-Fe^3+^ from the BL to the AP domain, and the release of bound Fe^3+^ directly to the neuroretina^36^ for conjugation with retinal Tf that is distinct from plasma Tf, and is synthesized locally by cells of the neuroretina^17, 36–38^. However, conclusive evidence for either of the above models is lacking. Our data shows significant upregulation of ferritin and down-regulation of TfR and DMT-1 upon polarization of RPE cells, indicating that polarization promotes iron uptake, and these cells regulate retinal iron homeostasis by down regulating iron uptake proteins based on cytosolic ferritin levels as reported for the BBB and other cell types^6, 39, 40^. The mechanism by which iron is exported from the AP domain of RPE cells to the neuroretina remains unclear.

Several observations from our data indicate that PrP^C^ facilitates uptake of iron by RPE cells, thereby modulating retinal iron homeostasis. *In vivo* data in support of this assumption is based on ferritin levels in the neuroretina, which were significantly lower in PrP^−/−^ samples relative to controls. Although systemic iron overload increased neuroretinal iron in both mouse lines, uptake of ^59^Fe and ferritin levels remained lower in PrP^−/−^ samples, indicating that other ferrireductase proteins including Steap3 and Dcytb^33^ are unable to compensate for the absence of PrP^C^. It is notable that up regulation of ferritin in iron-overloaded mice was mainly in the RPE cell layer, supporting their role in regulating iron uptake in addition to serving as a conduit for transport^40, 41^. However, despite the stringent outer and inner blood-retinal barriers, a significant amount of NTBI was transported to the neuroretina, supporting a previous report^42^, and explaining the beneficial effect of metal chelators^42, 43^. Interestingly, PrP^C^ is cleaved at the β-site by redox-active iron that results in the loss of its ferrireductase domain, suggesting that in addition to its facilitative role, PrP^C^ regulates iron uptake (discussed below).

Our observations on the RPE cell line suggest that PrP^C^ provides the ferrireductase activity necessary for the transport of iron through DMT-1. Thus, down regulation of PrP^C^ reduced cellular ferritin, while over-expression up regulated ferritin levels. This effect was amplified by extracellular iron, which, in addition, resulted in β-cleavage of PrP. Whether PrP^C^ functions as a ferrireductase on the BL membrane or in the endosomes of RPE cells is unclear from our data. This question is of particular importance in the context of NTBI that is reduced on the plasma membrane before transport to the cytosol through divalent metal transporters such as DMT-1 and the ZIP family of proteins^44^. Further exploration is necessary to clarify this question.

As mentioned above, almost 90% of PrP^C^ is cleaved at the β-site in the neuroretina *in vivo* and in polarized RPE cells *in vitro*, an unexpected observation since in most cells PrP^C^ is cleaved at the α-site during recycling from the plasma membrane. This event is mediated by a disintegrin and metalloproteinase (ADAMs 8, 10, 17)^45^. α-Cleavage disrupts the amyloidogenic and neurotoxic region of PrP^C^ comprising of amino acids 106–126, and releases a soluble N-terminal fragment N1 that is believed to be neuroprotective^26^. The truncated C-terminal fragment C1 is transported back to the plasma membrane, and is resistant to conversion to the PrP^Sc^ form. Additional cleavage sites described for PrP^C^ include β-cleavage that takes place at multiple sites within and adjacent to the octapeptide repeat region, and a recently described ɣ-cleavage site^27, 46^. β-Cleavage spares the amyloidogenic region of PrP^C^, and releases corresponding fragments N2 and C2. Unlike C1, C2 is permissive for conversion to the PrP^Sc^ form, and is often detected in diseased brains. Cleavage at the β-site is believed to be mediated by calpains in the cytosol^47^ or cathepsins in the lysosomal compartment^28^, though the bulk of evidence points towards oxidative stress as the principal mediator of this cleavage^48, 49^. The released N-terminal fragment N2 is believed to protect the cells from reactive oxygen species (ROS)^48, 50^. The presence of mainly β-cleaved form of PrP^C^ in RPE cells suggests that either these cells lack the enzymes that cleave PrP^C^ at the α-site, or the metabolic processes specific to these cells favor cleavage at the β-site. Based on our observations, it is likely that β-cleavage occurs in conjunction with the reduction of Fe^3+^ to Fe^2+^. The octapeptide repeat region of PrP^C^ is known to reduce copper^51–54^, and exposure to copper is known to cause extensive β-cleavage of PrP^C^ under reducing conditions^55^. It is likely that a similar cleavage occurs in acidic recycling endosomes where Fe^3+^ is released from Tf and reduced to Fe^2+^ ^56^. Together, these observations support our hypothesis that reduction of iron and β-cleavage of PrP^C^ are coupled, and the release of N2 fragment serves a regulatory role by removing the FR domain of PrP^C^.

Relatively little accumulation of immunoreactive PrP^Sc^ plaques despite extensive degeneration of the neuroretina during the clinical stage of scrapie infection was surprising. Moreover, there was little, if any, accumulation of PrP^Sc^ in the RPE cell layer despite abundant presence of β-cleaved PrP^C^ that is amenable to conversion. However, upregulation and aggregation of ferritin was especially prominent in the RPE cell layer. The accompanying microglial activation suggests that inflammation is an integral component of PrP^Sc^ infection, and is partly responsible for the cytotoxicity and iron imbalance observed in the infected neuroretina. The former is likely to be mediated by pro-inflammatory effector cytokines^57, 58^, and the latter as a result of down-regulation of the iron export protein Fpn mediated by hepcidin, a peptide hormone released in response to inflammation^59^. Such a response is likely to induce a phenotype of iron deficiency much like the anemia of chronic disease, and exacerbate neuroretinal degeneration due to functional iron deficiency. Aggregation of ferritin in sCJD brains has been reported earlier, and attributed to co-aggregation with PrP^Sc^ in lysosomal compartments^12, 13^. However, the disproportionate accumulation and aggregation of ferritin relative to PrP^Sc^ especially in the RPE cell layer suggests sequestration of iron in ferritin in response to inflammation. Since Fpn is expressed on the BL membrane of RPE cells, it is likely that its downregulation due to inflammation leads to the accumulation of iron and upregulation of ferritin especially in these cells. The low pH of the lysosomal compartment is likely to induce its aggregation, sequestering iron in a biologically unavailable form. Upregulation of Tf is probably in response to chronic iron deficiency induced by PrP^Sc^ infection, worsening the iron imbalance and associated cytotoxicity.

In conclusion, this study demonstrates that PrP^C^ mediates iron uptake by the neuroretina, and is cleaved at the β-site during this process. Moreover, chronic inflammation as a result of PrP^Sc^ accumulation induces functional iron deficiency, contributing to prion disease associated neurotoxicity. Since iron is sequestered in aggregated ferritin, it is unlikely that systemic or local chelation of iron will resolve this phenotype. Further exploration is necessary to resolve this complex matter.

## Methods

### Animals and ethics statement

Animals were housed in the Case Western Reserve University (CWRU) School of Medicine animal resource center. The animal protocol was reviewed and approved in accordance with provisions of the Animal Welfare Act and Guide for the Care and Use of Laboratory Animals, as well as U.S. Government Principles for the Utilization and Care of Vertebrate Animals Used in Testing, Research, and Training. The animals were observed daily for signs of illness by the animal technician responsible for husbandry. The Case PHS Assurance number is A-3145–01, valid till 04/30/19. The animal protocols relevant to this study (#2015–0027 and 2014–0070) were approved by the IACUC committee at CWRU. All experiments were carried out in accordance with the above guidelines and regulations. The animals were kept under a 12 hour day-night cycle and had ad libitum access to food and water.

Wild-type (PrP^+/+^) and PrP knock-out (PrP^−/−^) mice were originally obtained from George Carlson, and bred on the FVB/NJ background for more than 15 generations at our facility. Homozygous transgenic mice expressing human PrP on the FVB/NJ PrP^−/−^ background (TgHu-PrP) were derived from our Tg40 mice^23^. Syrian hamsters were obtained from Jackson laboratories and housed in the same facility during the course of the experiment.

The FVB/NJ strain of mice carry the *Pde6b* ^*rd1*^ mutation that results in almost complete degeneration of rod photoreceptors by the time the mice are 1 month old. All other retinal cell types are preserved^60, 61^. Immunohistochemical analysis of retinal sections from PrP^+/+^ and PrP^−/−^ mice ranging in age from 3–21 days confirmed this phenotype (Supplementary Fig. 1), and was considered in planning the experimental design. Accordingly, to harvest all layers of the neuroretina, 9–13 day old mice were used. To analyze mainly the RPE cell layer, samples from 1–2 month old mice were used.

### Antibodies and chemicals

PrP^C^-specific antibodies 3F4 and 8H4 were obtained from Signet laboratories (Dedham, MA, USA). 3F4 binds to an epitope on amino acids 109–112 (Human PrP sequence), while 8H4 binds an epitope in the C-terminal amino acids 145–180 of human and mouse PrP^62^. Other primary antibodies were obtained from the following sources: anti-ferritin (F5012, Sigma Aldrich, USA), anti-DMT-1 (ab55812, Abcam, USA), anti-TfR (ab84036, Abcam, USA), anti-Tf (GTX21223, GeneTex, USA), and anti-β-actin (MAB1501, Millipore, USA) Secondary antibodies conjugated with HRP were from GE Healthcare (anti-mouse, LNA931V, anti-rabbit, LNA934V). Alexa fluor-conjugated secondary antibodies were from molecular probes, ImmPACT DAB (SK4105) from Vector labs, USA, PNGase F (P0704S) from NEB, USA and Lipofectamine 3000 transfection reagent from Invitrogen, USA. Radiolabeled ^59^FeCl_3_ was obtained from Perkin-Elmer, USA. siRNA against PrP (sc36318) and scrambled siRNA (sc37007) were purchased from Santa Cruz Biotechnology Inc, USA. For immunohistochemistry of hamster retinal sections PrP-6C2 (CVI-WUR, Lelystad, Netherland), ferritin (Jackson ImmunoResearch, West Grove, PA, USA), and Iba1 (019–19741, Wako Chemicals, USA) antibodies were used. FAC (F5879) and all other chemicals were from Sigma Aldrich, USA.

### Cell lines, expression constructs, RNAi silencing

The human retinal pigment epithelial cell line RPE19 was obtained from ATCC and maintained in high glucose DMEM supplemented with 10% FBS at 37 °C in a humidified atmosphere with 5% CO_2_. The neuroblastoma cell line M17 has been described previously^63^. Plasmid constructs expressing PrP^C^ in piggyBac vector have been described^25^. Stable clones of RPE cells expressing PrP^C^ or vector were used for all experiments. To obtain polarized monolayers, RPE cells at a density of 1.5 × 10^5^/ml were seeded on 0.4 µm pore size trans-well inserts (Corning costar, Tewksbury, MA) in DMEM supplemented with 10% FBS. After one day, the medium was changed to DMEM-F12 with 1% FBS in both chambers. Monolayers were maintained till trans-epithelial resistance (TEER) of 140 Ω-cm^2^ or more was obtained. For silencing with RNAi, non-polarized RPE19 cells were transfected with siRNA against PrP^C^ and scrambled control using Lipofectamine 3000 (Invitrogen, USA). Lysates were prepared after 72 h and subjected to Western blotting.

### ^59^Fe uptake *in vivo*

Age and sex-matched PrP^+/+^ and PrP^−/−^ mice were injected with ^59^FeCl_3_ intraperitoneally prior to or following the administration of 70 µg/22 g mouse weight of FAC. The former radiolabels plasma Tf (^59^Fe-Tf), whereas the latter radiolabels mainly NTBI^24^. Both sets of mice were euthanized after 24 hours, infused with PBS to flush out capillary blood, rinsed in PBS, and uptake of ^59^Fe was quantified in a γ-counter (Beckman Gamma 5500). Subsequently the posterior segment was dissected and exposed to X-ray film to generate an autoradiogram.

### Fe uptake *in vitro*

RPE cells stably expressing vector or PrP^C^ were supplemented with 30 µM FAC overnight, and lysates were processed for evaluation by western blotting. Protein lysates were deglycosylated with PNGase-F (NEB, USA), where indicated.

### Immunohistochemistry of scrapie-infected eyes

Four week old female hamsters were inoculated with scrapie by the intracerebral (ic) route (50 µl, 1% 263K-infected hamster brain homogenate in PBS), and sacrificed at 49 and 75 days post-inoculation and at the clinical stage when ataxia, head tilting, and ruffled coat were prominent (~80 days pi). Matched set of animals inoculated with uninfected brain homogenate were used as negative controls (Mock). At the indicated times, experimental and control animals were euthanized, and enucleated eyes were processed for Western blotting (see below) or fixed in Bouin’s fixative for 24 hours and post-fixed in 10% formalin until processing. Posterior segment of each eye was embedded in paraffin, and 4 μm sections of mock and scrapie-infected samples were rehydrated with xylene, followed by a decreasing ethanol concentration gradient (100%, 90% 70%) and a final wash with diH_2_O. Heat-mediated antigen retrieval was performed using EDTA buffer (10 mM Trizma Base, 1 mM EDTA solution, 0.05% Tween 20, pH 9.0) in an autoclave for 30 minutes. Tissues were then incubated with Background Buster (Innovex Biosciences Inc., Richmond, CA, USA) for 1.5 hours. Primary antibody against PrP (6C2; CVI-WUR, Lelystad, Netherlands) was diluted 1:2000, against ferritin 1:200 (Jackson ImmunoResearch, West Grove, PA, USA), and against Iba1 1:500 (Wako Chemicals, USA) in blocking solution containing 0.1% bovine serum albumin (BSA; Sigma-Aldrich, St. Louis, MO), 0.05% Triton X-100 (Thermo Fisher Scientific, Inc., Rockford, IL, USA), and 5% normal donkey serum (Jackson ImmunoResearch, West Grove, PA, USA) in TBS. The sections were incubated for 48 hours at room temperature and then 24 hours at 4 °C. Tissues were washed with TBS-T (6 × 5 minutes), and immunoreactivity for PrP was developed using Envision þ Dual Link System-horseradish peroxidase (Dako Corp., USA) with diaminobenzidene (Vector Labs, Peterborough, UK), and slides were counterstained with hematoxylin. For immunofluorescence detection of Iba1 and ferritin reactivity, sections were incubated with Cy^TM^3 conjugated AffiniPure secondary antibody (1:250; Jackson ImmunoResearch, West Grove, PA, USA) and 40,6-diamidino-2phenylindole dilactate (DAPI, 1:50; Sigma-Aldrich, USA) for 1.5 hours. Following another wash, slides were mounted with Vectashield Hard Set antifade mounting medium (Vector Laboratories Inc., Burlingame, CA, USA). Negative controls were processed in parallel by omission of the primary and/or secondary antibody. Fluorescence images were captured at 20x, and 63x, using a commercial upright microscope system (Zeiss AxioPlan 2 Microscope Imaging System; Oberkochen, Germany).

### Isolation of neuroretina

Enucleated eyes were washed in PBS and the surrounding connective tissue and optic nerve was removed. Subsequently, the eyeball was incubated in 1% dispase at 37 °C for 45 min, and posterior segment was separated from rest of the structures. The retina was scraped off under a light dissecting microscope. Isolated tissue was solubilized in lysis buffer, and processed for Western blotting as described^64^.

### SDS-PAGE and Western Blotting

Cell or tissue samples were lysed in buffer containing 100 mM NaCl, 20 mM Tris-HCl, pH 7.4, 1% NP-40, 0.5% DOC and 10 mM EDTA. Samples were clarified by centrifugation, subjected to deglycosylation with PNGase-F (NEB, USA), fractionated by SDS-PAGE, and subjected to Western blotting. To isolate detergent-insoluble PrP^Sc^, retinal lysates from 6 hamster eyes were mixed, and centrifuged at 18,000 g. The detergent soluble and insoluble phases were fractionated by SDS-PAGE followed by Western blotting. Transferred proteins were probed with antibodies specific for PrP (3F4, 1:1000; anti-C or 8H4, 1:500 (Signet laboratories, Dedham, MA, USA), ferritin,1:1000 (Sigma Aldrich, USA), TfR, 1:2000 (Abcam, USA), Tf, 1:1000 (GeneTex, USA), DMT-1,1:1000 (Abcam, USA), β-actin, 1:10000 (Millipore, USA), anti-mouse-HRP, 1:15000 (GE Healthcare, USA), anti-rabbit-HRP, 1:15000 (GE Healthcare, USA). Protein bands were quantified by densitometry using UN-SCAN-IT (version 6.1) software (Silk Scientific) and graphically presented using GraphPad Prism (version 5.0) software (GraphPad Software Inc.).

### Statistical analysis

All statistical analysis was done using GraphPad Prism (Version 5) Software (GraphPad Software, Inc, La Jolla, CA). Data are represented as Mean ± Standard error of mean (SEM). Level of significance was calculated by unpaired student’s t-test between control and experimental group.

## Electronic supplementary material

Supplementary file
