# Supplementary material for: Prion protein facilitates retinal iron uptake and is cleaved at the β-site: Implications for retinal iron homeostasis in prion disorders
Source: Sci Rep. 2017 Aug 29;7:9600. doi: 10.1038/s41598-017-08821-1 (PMC5575325; doi:10.1038/s41598-017-08821-1)
Supplement: Supplementary file 1 — Supplementary file [file 41598_2017_8821_MOESM1_ESM.pdf]

Prion protein facilitates retinal iron uptake and is cleaved at the β-site: Implications for retinal iron homeostasis in prion disorders

Abhishek Asthana[1], Shounak Baksi[1], Ajay Ashok[1], Shilpita Karmakar[1], Najiba Mammadova[2], Robyn Kokemuller[2], Mary Heather Greenlee[2], Qingzhong Kong[1], Neena Singh[1]*

***Corresponding author**
E-mail: neena.singh@case.edu

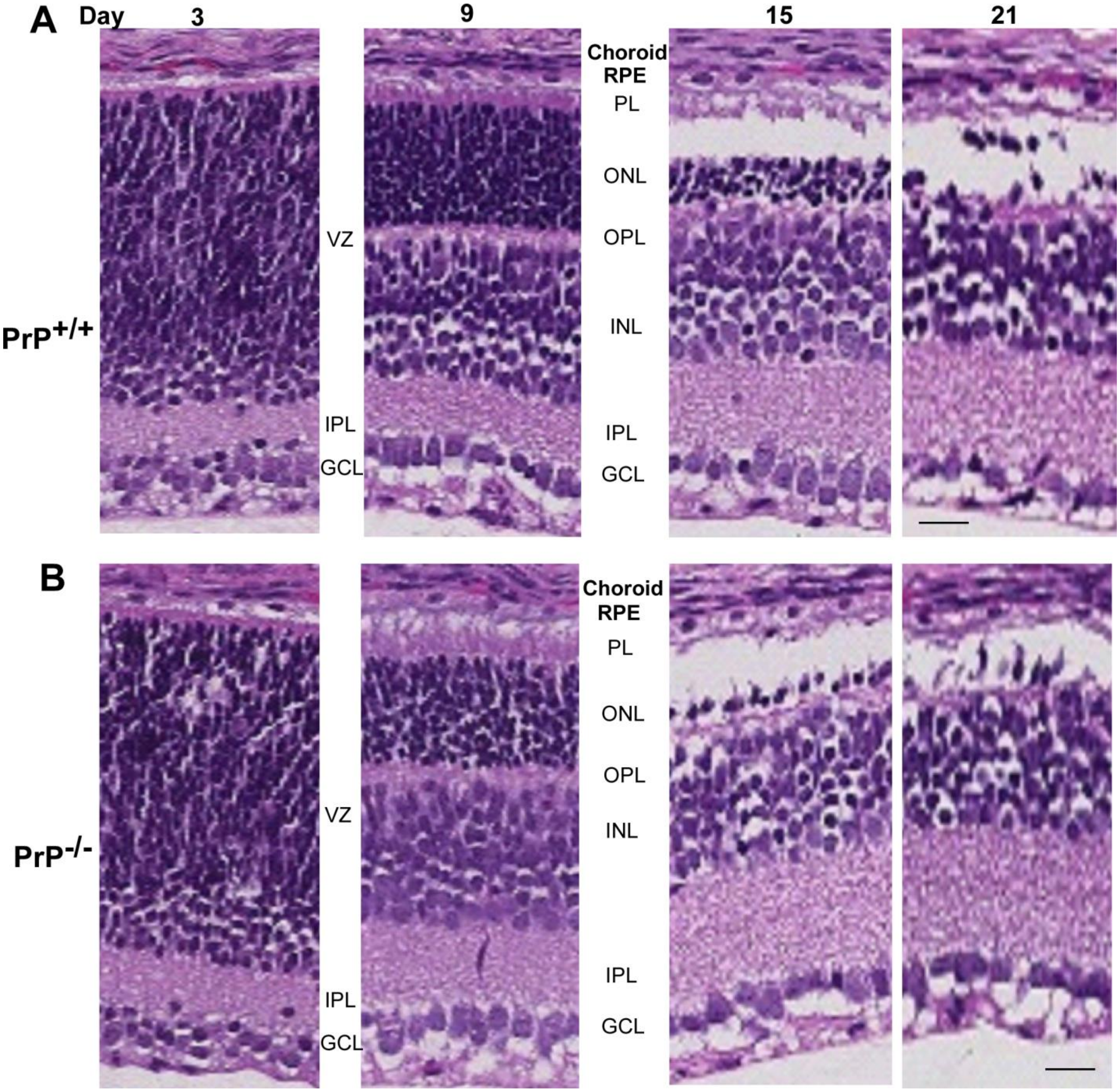

Supplementary Figure. Change in retinal morphology in FVB/NJ mice with age. Retinal sections harvested from FVB/NJ wild-type ($PrP^{+/+}$) and $PrP^{-/-}$ mice on post-natal day 3, 9, 15 and 21 were stained with H&E and examined. All layers of the retina were easily identifiable till day 9, after which gradual degeneration of the rod photoreceptors began, and was almost complete by day 21. RPE: retinal pigment epithelium; VZ: ventricular zone; PL: photoreceptor layer; ONL: outer nuclear layer; OPL: outer plexiform layer; INL: inner nuclear layer; IPL: inner plexiform layer; GCL: ganglion cell layer. Bar: 20µm.

Note: Post-natal day 3 retina has 3 separate layers GCL, IPL and a thick layer of neuroblasts in various stage of mitosis referred as the ventricular zone (VZ). On day 9, all retinal layers are easily discernable, and reach the adult morphology by day 25. However FVB/NJ mice are homozygous for the $Pde6b^{rd1}$ mutation that results in degeneration of the photoreceptor cell layer. All other layers are normal [1].

Reference

1 Yang, J., Nan, C., Ripps, H. & Shen, W. Destructive Changes in the Neuronal Structure of the FVB/N Mouse Retina. *PLoS One* **10**, e0129719 (2015).
